# Ontario Veterinary Alumni of Massachusetts

**Published:** 1897-11

**Authors:** Francis Abele

**Affiliations:** Secretary, Quincy, Mass.


### ONTARIO VETERINARY ALUMNI OF MASSACHUSETTS.

A meeting was held at the United States Hotel in Boston, October 7th, by a number of graduates of the Ontario Veterinary College, and there organized into the "Ontario Veterinary Alumni of Massachusetts," with Dr. Pierce, of Boston, President; Dr. Keene, Fitchburg, Treasurer, and Dr. Francis Abele, of Quincy, Secretary.

A paper was read by Dr. Abele on "Parturient Apoplexy;" another by Dr. Pierce, on "Thrombus of the Metacarpal Artery." Considerable discussion and exchange of ideas resulted. At the next meeting, which is to be held at the same place on the first Wednesday of December, papers will be read by Drs. Keene and Stearns.

The Society would like to learn of any Ontario graduates in the State with whom it has not corresponded. Such should correspond with Dr. Abele, of Quincy.

Dr. Francis Abele,
Secretary, Quincy, Mass.

